# Effects of sport-related repetitive subconcussive head impacts on biofluid markers: a scoping review protocol

**DOI:** 10.1136/bmjopen-2020-046452

**Published:** 2021-06-28

**Authors:** Liivia-Mari Lember, Michail Ntikas, Stefania Mondello, Lindsay Wilson, Angus Hunter, Thomas Di Virgilio, Emanuela Santoro, Magdalena Ietswaart

**Affiliations:** 1 Department of Psychology, University of Stirling Faculty of Natural Sciences, Stirling, UK; 2 Biomedical and Dental Sciences and Morphofunctional Imaging, University of Messina Faculty of Medicine and Surgery, Messina, Italy; 3 Physiology Exercise and Nutrition Research Group, University of Stirling Faculty of Health Sciences and Sport, Stirling, UK

**Keywords:** neurological injury, neuropathology, sports medicine

## Abstract

**Introduction:**

Sport-related repetitive subconcussive head impacts (RSHIs) are increasingly thought to be associated with adverse long-term outcomes. However, owing to potentially subtle effects, accurate assessment of harm to the brain as a consequence of RSHI is a major challenge and an unmet need. Several studies suggest that biofluid markers can be valuable objective tools to aid the diagnosis and injury characterisation and help in medical decision-making. Still, by and large, the results have been limited, heterogeneous and inconsistent. The main aims of this scoping review are therefore (1) to systematically examine the extent, nature and quality of available evidence from studies investigating effects of RSHI on fluid biomarkers and (2) to formulate guidelines and identify gaps with the aim to inform future clinical studies and the development of research priorities.

**Methods and analyses:**

We will use a comprehensive search strategy to retrieve all available and relevant articles in the literature. The following electronic databases will be systematically searched: MEDLINE (EBSCO host; from 1809 to 2020); Scopus (from 1788 to 2020); SPORTDiscus (from 1892 to 2020); CINAHL Complete (from 1937 to 2020); PsycINFO (from 1887 to 2020); Cochrane Library (to 2020); OpenGrey (to 2020); ClinicalTrials.gov (to 2020) and WHO International Clinical Trials Registry Platform (to 2020). We will consider primarily biomedical studies evaluating the biofluid markers following RSHI. Two independent reviewers will screen articles for inclusion using predefined eligibility criteria and extract data of retained articles. Disagreements will be resolved through consensus or arbitrated by a third reviewer if necessary. Data will be reported qualitatively given the heterogeneity of the included studies. In synthesising the evidence, we will structure results by markers, sample types, outcomes, sport and timepoints.

**Ethics and dissemination:**

Ethics approval is not required. We will submit results for peer-review publication, and present at relevant conferences.

**Strengths and limitations of this study**

- To our knowledge, this scoping review is the first review to systematically and comprehensively map the existing evidence of the effects of repetitive subconcussive head impact (RSHI) on biofluid markers.
- We employ broad inclusion criteria to ensure that relevant studies are not missed.
- Results from our study are designed to advance the understanding of the effects of RSHIs on neurobiochemical markers and provide guidelines for future clinical research.
- A potential limitation may be a lack of a universally accepted and standardised definition of what subconcussive head impacts are, which may introduce increased subjective judgement with regards to inclusion of studies.

## Introduction

Unlike concussion, subconcussive head impacts do not frequently elicit overt symptoms and, therefore, are often regarded as benign. Nonetheless, there is emerging evidence that both concussive and subconcussive impacts result in structural and functional changes in the brain,^1 2^ representing a potential contributing factor to long-term cognitive sequalae and/or neurodegenerative disease.^3–5^ This raises concerns for athletes participating in contact sports who routinely experience repetitive head impacts, particularly owing to the challenge to accurately detect resulting subtle brain changes.

Measurable biomarkers in blood have been found to significantly increase following traumatic brain injury (TBI),^6–8^ and be correlated with injury severity and outcomes.^9 10^ Hence, they might be a helpful diagnostic tool,^11 12^ capable of informing management of athletes. However, studies evaluating biomarker concentrations following repetitive subconcussive head impacts (RSHIs) such as in contact sport athletes have yielded mixed results.^13–19^ These contrasting findings may be caused by methodological and analytical variability (eg, diverse settings, sampling times and types, and study designs) as well as the lack of a universally accepted and standardised definition of RSHI. For the purposes of this review, RSHIs are operationally defined as routine repetitive intentional or unintentional non-concussive head impacts acquired during contact sport participation. On completion of this scoping review, the aim is to provide a comprehensive evidence-based operational definition of RSHI to be adopted in future work.

The use of biofluid markers for detection of brain changes following RSHI is an emerging field of research. As such, we considered that at this stage a scoping review comprehensively mapping the studies from different sports applying various designs will be more advantageous compared with a systematic review that would be restricted to only part of the existing literature.^20 21^ Therefore, the aim of this review is to systematically scope the existing body of evidence, evaluate the quality and the adequacy of reporting, and identify research gaps to guide future research to support the clinical utility of biomarkers for RSHI.

### Objectives

The primary aim is to systematically examine the extent, nature and quality of available studies that have investigated the effects of RSHI on biofluid brain injury markers. The secondary aim is to reach an informed view of an acceptable set of features for future work concerning RSHI, formulate guidelines for future research and identify literature gaps to inform future clinical studies and the development of research priorities. An additional aim is to assess the feasibility of conducting systematic review and meta-analysis investigating the effects of RSHI on biofluid markers.

## Methods

In this scoping review, we will use a systematic and comprehensive approach to retrieve all studies published in peer-reviewed academic journals, along with registered clinical trials and the grey literature. Our work will adhere to the Preferred Reporting Items for Systematic Reviews and Meta-Analyses extension for Scoping Reviews.^22^

### Study eligibility

This review will include studies where acute or chronic exposure to sport-related RSHIs occurred and where biofluid markers were assessed (including, but not limited to, S-100B, NF-L, T-tau, NSE, GFAP and microRNA). Studies with the primary aim to assess the effect of RSHI on biofluid markers will be included if there is evidence of RSHI exposure. This evidence could be minutes of boxing training, years of soccer playing, boxing/rugby matches played and so on. Studies primarily focused on concussion in which contact sport control groups were used, will be included if there is sufficient evidence of subconcussive head impact exposure in the control group (impact data and/or video recording) and biofluid marker samples were taken before and after this exposure, thus making it a valid experimental group for assessing RSHI.

This review will not include studies assessing biomarker concentrations following solely sports-related concussion or TBI or studies assessing biofluid markers of peripheral injury. We will also exclude any studies with potentially overlapping population/biomarker data and review articles.

We will not place any restrictions on methodological standards, design and sample size. Studies will be included regardless of geographic location and date of publication. We will be examining reports in the English, French, German and Italian languages.

### PECOS criteria

We will include clinical studies if they contain the Participants, Exposure, Comparisons, Outcomes, Setting (PECOS) criteria outlined below.

#### Types of participants

The population of interest in this review are active or retired male and female contact sport players (including but not limited to American football, rugby, ice-hockey, soccer and boxing) of any age and player level.

#### Types of interventions/exposure

Acute or chronic exposure to RSHI. Those impacts may be a result of either a direct head impact acquired through, for example, soccer heading, sparring and head-to-body collisions, or indirectly through full-body collisions between players or between player and object.

#### Types of comparisons

We will include all possible comparisons; studies with within and between groups/conditions designs are acceptable, as well as any type of control groups/conditions such as static or exercise-based control groups. Studies without control groups/conditions, as well as comparisons between exposure to high versus low number of impacts will also be included in this scoping review.

#### Type of outcome measures

The concentrations of biofluid markers following acute or chronic exposure to RSHI across groups/conditions and, where reported, the differences between the concentrations pre-to-post RSHI serve as outcome measures.

#### Setting

We will include all relevant settings (eg, field and lab-based studies).

### Search strategy

An electronic search will be carried out in the following databases: Cochrane Library, CINAHL Complete, PsycINFO, MEDLINE (EBSCO host), Scopus, SPORTDiscus and OpenGrey. The following databases will be searched for ongoing registered clinical trials: ClinicalTrials.gov, WHO International Clinical Trials Registry Platform. Reference lists of included studies will also be searched.

Key descriptors that include terms for subconcussive head impacts, biomarker, contact sport, will be used for the search (see table 1).

**Table 1:** Search strategy including the three concepts and corresponding keywords

Concept	Keywords
Subconcussive head impacts	(subconcussi*) OR (sub-concussi*) OR (concussi*) OR (*TBI) OR (trauma*) OR (impact*) OR (head*) OR (brain*) OR (injur*)
Biomarker	(biomarker*) OR (marker*) OR (cytokines) OR (coagulation) OR (blood) OR (plasma) OR (serum) OR (“cerebrospinal fluid”) OR (CSF) OR (saliva) OR (urine) OR (neurofilament) OR (NFL) OR (S100*) OR (S −100*) OR (tau) OR (enolase) OR (NSE) OR (“glial fibrillary acidic protein”) OR (GFAP) OR (glial) OR (microRNA*) OR (miRNA*) OR (UCH -L1) OR (“Ubiquitin C -terminal hydrolase L1”) OR (“Ubiquitin carboxyl -terminal hydrolase isozyme L1”)
Contact sport	(contact NEAR/1 sport*) OR (collision NEAR/1 sport*) OR (soccer) OR (football) OR (boxing) OR (sparr*) OR (rugby) OR (*hockey)

Concepts will be separated with the Boolean operator ‘AND’. Depending on the database minor adjustments in keywords and proximity operators will be applied.

### Study selection

After removal of duplicates, two reviewers will independently screen the titles and abstracts against the eligibility criteria. Any disputes between reviewers will be resolved through discussion and if necessary, by a third member. The same process will be repeated on the full text to confirm inclusion in the scoping review. Studies excluded during the full-text screening will be listed with exclusion reason(s) as an appendix. This information on exclusion reason(s) will also list whether any arbitration by a third judge was employed

### Data extraction

Data will be recorded independently by two reviewers using a standardised data collection form. Disagreements will be discussed until consensus is reached and, if necessary, a third reviewer will be consulted for arbitration. The following information will be extracted if possible: (1) first author, (2) year of publication, (3) title, (4) study aim(s), (5) study design, (6) participant characteristics (sample size, age, sex, sport and exclusion/inclusion criteria), (7) control group (within or between group/condition comparisons; control group characteristics—static, exercise, no impacts, some impacts but less than in the exposed group, etc), (8) setting (laboratory-based or field study), (9) characteristics of biomarkers (biomarker(s) investigated, time of sampling, sampling source (plasma/serum/cerebrospinal fluid/saliva), levels (mean±SD or median with IQR), any other data deemed relevant), (10) laboratory aspects (type of assay used, limit of detection, limit of quantitation and sampling to freezing time), (11) impact data (ie, source of impacts (eg, football heading) with the number of impacts, and linear and rotational acceleration (mean±SD or median with IQR), (12) method for impact recording (ie, accelerometer and gyroscope, video footage, self-report and estimation based on the previous literature), (13) outcome measures and findings (eg, cognitive or brain-related clinical measures) and (14) study limitations.

### Risk of bias and quality assessment of included studies

A modified version of ROBINS-I risk of bias assessment will be used in this review.^23^ Moreover, a modified version of the subconcussion-specific tool will be used to assess the quality of the included studies.^2 24^ Even though performing a risk of bias and quality assessment is not a prerequisite for a scoping review, its use will help improve the quality of this review and help determine acceptable features in the research domain of RSHI. Furthermore, the risk of bias and quality assessment can potentially provide evidence for the feasibility of future systematic review. ROBINS-I tool protocol stage is included in the online supplemental material S1.

10.1136/bmjopen-2020-046452.supp1 Supplementary data

### Data analysis

Studies will be grouped by the type of study (acute or chronic), biomarker and sport. Study findings, designs, populations, control groups, sources and amount of head impact exposure, sampling methods and setting will be summarised. Methodological differences, gaps in the literature and quality assessment of the included studies will be analysed to provide implications for future research, and to determine the advantages and feasibility of performing a further systematic review.

### Patient and public involvement

No new patients will be enrolled as part of this investigation.

## Discussion

This scoping review will provide an overview of the existing body of evidence of the effect of sport-related RSHIs on biofluid markers. Furthermore, the quality and limitations of the available evidence will be assessed to aid the development of high-quality future clinical studies by providing guidelines that will increase future studies’ clinical utility and homogeneity. Moreover, the findings of this review will inform the decision as to whether performing systematic review is feasible based on the existing body of evidence.

A strength of this review will be the comprehensive inclusion criteria and search strategy. However, the lack of a universally accepted and standardised definition of RSHIs may introduce ambiguity in the inclusion of studies. To minimise the risk of potential differences in opinion, this protocol has outlined the types of exposure that will be considered to qualify as RSHIs. Furthermore, we have designated a member of the research team to resolve any disputes that may occur. Moreover, the included studies will be assessed for quality, and limitations will be recorded. Reasons will be noted for studies that are excluded during the full-text screening.

Although the broad nature of scoping reviews will not allow for quantitative analysis,^21 22^ the findings from this work will be advantageous for future research by identifying the knowledge gaps and limitations within the emerging field of the effect of sport-related repetitive head impacts on biofluid markers.

## Ethics and dissemination

Ethics approval for this scoping review is not required. The findings will be published in a peer-reviewed journal and presented at relevant conferences, such as the Symposia of the National and International Neurotrauma Societies and the Neurocritical Care Society Meeting, and university and stakeholder workshops. Other forms of dissemination will include academic theses.

## Supplementary Material

Author's manuscript
