# Supplementary material for: Effects of sport-related repetitive subconcussive head impacts on biofluid markers: a scoping review protocol
Source: BMJ Open. 2021 Jun 28;11(6):e046452. doi: 10.1136/bmjopen-2020-046452 (PMC8240577; doi:10.1136/bmjopen-2020-046452)
Supplement: Author's manuscript [file bmjopen-2020-046452.draft_revisions.pdf]

BMJ Open is committed to open peer review. As part of this commitment we make the peer review history of every article we publish publicly available.

When an article is published we post the peer reviewers' comments and the authors' responses online. We also post the versions of the paper that were used during peer review. These are the versions that the peer review comments apply to.

The versions of the paper that follow are the versions that were submitted during the peer review process. They are not the versions of record or the final published versions. They should not be cited or distributed as the published version of this manuscript.

BMJ Open is an open access journal and the full, final, typeset and author-corrected version of record of the manuscript is available on our site with no access controls, subscription charges or pay-per-view fees (http://bmjopen.bmj.com).

If you have any questions on BMJ Open's open peer review process please email info.bmjopen@bmj.com

BMJ Open

Effects of Sport-related Repetitive Sub-concussive Head Impacts on Biofluid Markers: A Scoping Review Protocol

Journal:	*BMJ Open*
Manuscript ID	bmjopen-2020-046452
Article Type:	Protocol
Date Submitted by the Author:	18-Dec-2020
Complete List of Authors:	Lember, Liivia-Mari; University of Stirling Faculty of Natural Sciences, Department of Psychology<br>Ntikas, Michail; University of Stirling Faculty of Natural Sciences, Department of Psychology<br>Mondello, Stefania; University of Messina Faculty of Medicine and Surgery, Biomedical and Dental Sciences and Morphofunctional Imaging<br>Wilson, Lindsay; University of Stirling Department of Psychology,<br>Hunter, Angus; University of Stirling Faculty of Health Sciences and Sport, Physiology Exercise and Nutrition Research Group<br>Di Virgilio, Thomas; University of Stirling Faculty of Health Sciences and Sport, Physiology Exercise and Nutrition Research Group<br>Santoro, Emanuela; University of Messina Faculty of Medicine and Surgery, Biomedical and Dental Sciences and Morphofunctional Imaging<br>Donoghue, Emma; Monash University, School of Preventative Medicine and Public Health<br>Ietswaart, Magdalena; University of Stirling Faculty of Natural Sciences, Department of Psychology
Keywords:	Neurological injury < NEUROLOGY, Neuropathology < NEUROLOGY, SPORTS MEDICINE

SCHOLARONE™
Manuscripts

I, the Submitting Author has the right to grant and does grant on behalf of all authors of the Work (as defined in the below author licence), an exclusive licence and/or a non-exclusive licence for contributions from authors who are: i) UK Crown employees; ii) where BMJ has agreed a CC-BY licence shall apply, and/or iii) in accordance with the terms applicable for US Federal Government officers or employees acting as part of their official duties; on a worldwide, perpetual, irrevocable, royalty-free basis to BMJ Publishing Group Ltd ("BMJ") its licensees and where the relevant Journal is co-owned by BMJ to the co-owners of the Journal, to publish the Work in this journal and any other BMJ products and to exploit all rights, as set out in our licence.

The Submitting Author accepts and understands that any supply made under these terms is made by BMJ to the Submitting Author unless you are acting as an employee on behalf of your employer or a postgraduate student of an affiliated institution which is paying any applicable article publishing charge ("APC") for Open Access articles. Where the Submitting Author wishes to make the Work available on an Open Access basis (and intends to pay the relevant APC), the terms of reuse of such Open Access shall be governed by a Creative Commons licence – details of these licences and which Creative Commons licence will apply to this Work are set out in our licence referred to above.

Other than as permitted in any relevant BMJ Author's Self Archiving Policies, I confirm this Work has not been accepted for publication elsewhere, is not being considered for publication elsewhere and does not duplicate material already published. I confirm all authors consent to publication of this Work and authorise the granting of this licence.

For peer review only

Effects of Sport-related Repetitive Sub-concussive Head Impacts on Biofluid Markers: A Scoping Review Protocol

Liivia-Mari Lember*[1,2], Michail Ntikas*[1], Stefania Mondello[3], Lindsay Wilson[1], Angus Hunter[2], Thomas Di Virgilio[2], Emanuela Santoro[3], Emma Donoghue[4,] and Magdalena Ietswaart[1]

1. Department of Psychology, University of Stirling, Scotland, UK
2. Physiology Exercise and Nutrition Research Group, University of Stirling, Scotland, UK
3. Biomedical and Dental Sciences and Morphofunctional Imaging, University of Messina, Italy
4. School of Preventative Medicine and Public Health, Monash University, Melbourne, Australia

* joint first author

Corresponding author: Magdalena Ietswaart

Department of Psychology, University of Stirling

Stirling, FK9 4LA, United Kingdom

Email: magdalena.ietswaart@stir.ac.uk

Phone: + 44 (0) 7711001106

Word count: 1459

Keywords: scoping review, biomarkers, subconcussive head impacts, brain injury, diagnosis, sport

For peer review only

ABSTRACT

Introduction: Repetitive sub-concussive sport-related head impacts (RSHI) are increasingly thought to be associated with adverse long-term outcomes. However, owing to potentially subtle effects, accurate assessment of harm to the brain as a consequence of RSHI is a major challenge and an unmet need. Several studies suggested that biofluid markers can be valuable objective tools to aid the diagnosis and injury characterization and help in medical decision-making. Still, by and large, the results have been limited, heterogeneous and inconsistent. The main aims of this scoping review are therefore (1) to systematically examine the extent, nature, and quality of available evidence from studies investigating effects of RSHI on biomarkers, and (2) to formulate guidelines and identify gaps with the aim to inform future clinical studies and the development of research priorities.

Methods and analyses: We will use a comprehensive search strategy to retrieve all available and relevant articles in the literature. We will search the following electronic databases: Cochrane Library, CINAHL Complete, PsycINFO, MEDLINE (EBSCO host), Scopus, SPORTDiscus, OpenGrey, ClinicalTrials.gov and World Health Organization International Clinical Trials Registry Platform. We will consider primarily biomedical studies evaluating the biofluid markers following RSHI. Two independent reviewers will screen articles for inclusion using predefined eligibility criteria and extract data of retained articles. Disagreements will be resolved through consensus or arbitrated by a third reviewer if necessary. Data will be reported qualitatively given the heterogeneity of the included studies. In synthesizing the evidence, we will structure results by markers, sample types, outcomes, sport, and timepoints.

Ethics and dissemination: Ethics approval is not required. We will submit results for peer-review publication, and present at relevant conferences.

For peer review only

STRENGTHS AND LIMITATIONS OF THIS STUDY

- To our knowledge, this scoping review is the first review to systematically and comprehensively map the existing evidence of the effects of RSHI on biofluid markers.
- Results from our study are designed to advance the understanding of the effects of repetitive sub-concussive head impacts on neurobiochemical markers and provide guidelines for future clinical research.
- The inclusion criteria will not limit the studies assessed in this review.
- A potential limitation may be a lack of a universally accepted and standardized definition of what sub-concussive head impacts are, which may introduce increased subjective judgement with regards to inclusion of studies.

INTRODUCTION

Unlike concussion, sub-concussive head impacts do not frequently elicit overt symptoms and, therefore, are often regarded as benign. Nonetheless, there is emerging evidence that both concussive and sub-concussive impacts result in structural and functional changes in the brain,[1] representing a potential contributing factor to long-term cognitive sequalae and/or neurodegenerative disease.[2–4] This raises concerns for athletes participating in contact sports who routinely experience repetitive head impacts, particularly owing to the challenge to accurately detect resulting subtle brain changes.

Measurable biomarkers in blood have been found to significantly increase following traumatic brain injury (TBI),[5–7] and be correlated with injury severity and outcomes.[8,9] Hence, they might be a helpful diagnostic tool,[10,11] capable of informing management of athletes. However, studies evaluating biomarker concentrations following repetitive sub-

For peer review only

concussive head impacts (RSHI) in contact sport athletes have yielded mixed results.[12–18] These contrasting findings may be caused by methodological and analytical variability (e.g. diverse settings, sampling times and types, study designs) as well as the lack of a universally accepted and standardized definition of RSHI.

It is therefore essential to systematically scope the existing body of evidence, evaluate the quality and the adequacy of reporting, and identify research gaps to guide future research to support the clinical utility of biomarkers for RSHI.

Objectives

The primary aim is to systematically examine the extent, nature, and quality of available studies that have investigated the effects of RSHI on biofluid brain injury markers. The secondary aim is to formulate guidelines and identify gaps to inform future clinical studies and the development of research priorities. An additional aim is to assess the feasibility of conducting systematic review and meta-analysis investigating the effects of RSHI on biofluid markers.

METHODS

In this scoping review, we will use a systematic and comprehensive approach to retrieve all studies published in peer reviewed academic journals, along with registered clinical trials and grey literature. Our work will adhere to the PRISMA extension for scoping reviews.[19]

Study Eligibility

This review will include studies where acute or chronic exposure to repetitive sub-concussive sport-related head impacts occurred and where biofluid markers were assessed

For peer review only

(including, but not limited to, S-100B, NF-L, T-tau, NSE, GFAP, microRNA). Studies with the primary aim to assess the effect of RSHI on biofluid markers will be included even if they did not measure or estimate head impact exposure. Studies primarily focused on concussion in which contact sport control groups were used, will be included if there is sufficient evidence of sub-concussive head impact exposure in the control group (impact data, video recording) and biofluid marker samples were taken before and after this exposure, thus making it a valid experimental group for assessing RSHI.

This review will not include studies assessing biomarker concentrations following solely sports-related concussion or traumatic brain injury or studies assessing biofluid markers of peripheral injury. We will also exclude any studies with potentially overlapping population/biomarker data, and review articles.

We will not place any restrictions on methodological standards, design, sample size, geographic location, date of publication, examining reports in the English, French, German, and Italian language.

PECOS Criteria

We will include clinical studies if they contain the PECOS criteria outlined below:

Types of Participants

The population of interest in the current review are active or retired male and female contact sport players (including but not limited to American football, rugby, ice-hockey, soccer, boxing) of any age and player level.

For peer review only

Types of Interventions/Exposure

Acute or chronic exposure to RSHI. Those impacts may be a result of either a direct head impact acquired through for example soccer heading, sparring and head-to-body collisions, or indirectly through full-body collisions between players or between player and object.

Types of Comparisons

We will include all possible comparisons; studies with within and between groups/conditions designs are acceptable, as well as any type of control groups/conditions such as static or exercise-based control groups. Studies without control groups/conditions, as well as comparisons between exposure to high versus low number of impacts will also be included in this scoping review.

Type of Outcome Measures

The concentrations of biofluid markers following acute or chronic exposure to RSHI across groups/conditions and, where reported, the differences between the concentrations pre-to post RSHI serve as outcome measures.

Setting

We will include all relevant settings (for example field and lab-based studies).

Search Strategy

An electronic search will be carried out in the following databases: Cochrane Library, CINAHL Complete, PsycINFO, MEDLINE (EBSCO host), Scopus, SPORTDiscus, OpenGrey.

For peer review only

Following databases will be searched for ongoing registered clinical trials: ClinicalTrials.gov, World Health Organization International Clinical Trials Registry Platform. Reference lists of included studies will also be searched.

Key descriptors that include terms for Sub-concussive head impacts, Biomarker, Contact sport, will be used for the search. Supplemental material S1 shows the detailed search terms used in this scoping review.

Study Selection

After removal of duplicates, two reviewers will independently screen the titles and abstracts against the eligibility criteria. Any disputes between reviewers will be resolved through discussion and if necessary, by a third member. The same process will be repeated on the full text to confirm inclusion in the scoping review. Studies excluded during the full-text screening will be attached with exclusion reason(s) as an appendix.

Data Extraction

Data will be recorded independently by two reviewers using a standardized data collection form. Disagreements will be discussed until consensus is reached and, if necessary, a third reviewer will be consulted for arbitration. The following information will be extracted if possible: (1) First author, (2) Year of publication, (3) Title, (4) Study aim(s), (5) Study design, (6) Participant characteristics (sample size, age, sex, sport, exclusion/inclusion criteria), (7) Control group (within or between group/condition comparisons; control group characteristics – static, exercise, no impacts, some impacts but less than in the exposed group etc.), (8) Setting (laboratory based or field study), (9) Characteristics of biomarkers (biomarker(s) investigated, time of sampling,

For peer review only

sampling source [plasma/serum/CSF/saliva], levels [mean ± SD or median with IQR], any other data deemed relevant), (10) Laboratory aspects (type of assay used, limit of detection, limit of quantitation, sampling to freezing time), (11) Impact data (i.e., source of impacts [e.g. football heading] with the number of impacts, and linear and rotational acceleration [mean ± SD or median with IQR], (12) Method for impact recording (i.e. accelerometer and gyroscope, video footage, self-report, estimation based on previous literature), (13) Outcome measures and findings (e.g. cognitive or brain-related clinical measures), and (14) Study limitations.

Risk of Bias and Quality Assessment of Included Studies

A modified version of ROBINS-I risk of bias assessment will be used in this review.[20] Moreover, a modified version of the Subconcussion-Specific Tool (SST) will be used to assess the quality of the included studies.[21,22] Even though performing a risk of bias and quality assessment is not a prerequisite for a scoping review, its use will help improve the quality of this review and it can potentially provide evidence for the feasibility of future systematic review. ROBINS-I tool protocol stage is included in the supplementary material (S2).

Data Analysis

Studies will be grouped by the type of study (acute or chronic), biomarker, and sport. Study findings, designs, populations, control groups, sources and amount of head impact exposure, sampling methods and setting will be summarised. Methodological differences, gaps in the literature and quality assessment of the included studies will be analysed to provide implications for future research, and to determine the advantages and practicability of performing a further systematic review.

For peer review only

Patient and public involvement

No patient involved.

DISCUSSION

This scoping review of the effect of sport-related repetitive sub-concussive head impacts on biofluid markers will provide an overview of the existing body of evidence, its quality and limitations, and aid the development of high-quality future clinical studies by providing guidelines that will increase future studies' clinical utility and homogeneity. Moreover, the findings of this review will inform the decision as to whether performing systematic review is feasible based on the existing body of evidence.

DISSEMINATION AND CONCLUSIONS

The findings will be published in a peer-reviewed journal and presented at relevant conferences, such as the Symposia of the National and International Neurotrauma Societies and the Neurocritical Care Society Meeting, and university and stakeholder workshops. Other forms of dissemination will include academic theses.

REFERENCES

1 Di Virgilio TG, Hunter A, Wilson L, *et al.* Evidence for Acute Electrophysiological and Cognitive Changes Following Routine Soccer Heading. *EBioMedicine* 2016;**13**:66–71. doi:10.1016/j.ebiom.2016.10.029

2 Maas AIR, Menon DK, Adelson PD, *et al.* Traumatic brain injury: integrated approaches

For peer review only

to improve prevention, clinical care, and research. *Lancet Neurol* 2017;**16**:987–1048. doi:10.1016/S1474-4422(17)30371-X

3 McKee AC, Daneshvar DH, Alvarez VE, *et al.* The neuropathology of sport. *Acta Neuropathol* 2014;**127**:29–51. doi:10.1007/s00401-013-1230-6

4 Wilson L, Stewart W, Dams-O'Connor K, *et al.* The chronic and evolving neurological consequences of traumatic brain injury. *Lancet Neurol* 2017;**16**:813–25. doi:10.1016/S1474-4422(17)30279-X

5 Herrmann M, Curio N, Jost S, *et al.* Protein S-100B and neuron specific enolase as early neurobiochemical markers of the severity of traumatic brain injury. *Restor Neurol Neurosci* 1999;**14**:109–14.

6 Shahim P, Tegner Y, Wilson DH, *et al.* Blood biomarkers for brain injury in concussed professional ice hockey players. *JAMA Neurol* 2014;**71**:684–92. doi:10.1001/jamaneurol.2014.367

7 Shahim P, Tegner Y, Marklund N, *et al.* Neurofilament light and tau as blood biomarkers for sports-related concussion. *Neurology* 2018;**90**:E1780–8. doi:10.1212/WNL.0000000000005518

8 Mondello S, Guedes VA, Lai C, *et al.* Sex Differences in Circulating T-Tau Trajectories After Sports-Concussion and Correlation With Outcome. *Front Neurol* 2020;**11**:1–8. doi:10.3389/fneur.2020.00651

9 Mondello S, Guedes VA, Lai C, *et al.* Circulating Brain Injury Exosomal Proteins following Moderate-To-Severe Traumatic Brain Injury: Temporal Profile, Outcome Prediction and Therapy Implications. *Cells* 2020;**9**:1–12. doi:10.3390/cells9040977

10 Gill J, Latour L, Diaz-Arrastia R, *et al.* Glial fibrillary acidic protein elevations relate to

For peer review only

neuroimaging abnormalities after mild TBI. *Neurology* 2018;**91**:E1385–9. doi:10.1212/WNL.0000000000006321

11 Mondello S, Sorinola A, Czeiter E, *et al.* Blood-Based Protein Biomarkers for the Management of Traumatic Brain Injuries in Adults Presenting to Emergency Departments with Mild Brain Injury: A Living Systematic Review and Meta-Analysis. *J Neurotrauma* 2018;**21**:1–21. doi:10.1089/neu.2017.5182

12 Dorminy M, Hoogeveen A, Tierney RT, *et al.* Effect of soccer heading ball speed on S100B, sideline concussion assessments and head impact kinematics. *Brain Inj* 2015;**29**:1158-1164. doi:10.3109/02699052.2015.1035324

13 Kawata K, Rubin LH, Wesley L, *et al.* Acute Changes in Plasma Total Tau Levels Are Independent of Subconcussive Head Impacts in College Football Players. *J Neurotrauma* 2018;**35**:260–6. doi:10.1089/neu.2017.5376

14 Rubin LH, Tierney R, Kawata K, *et al.* NFL blood levels are moderated by subconcussive impacts in a cohort of college football players. *Brain Inj* 2019;**33**:456–62. doi:10.1080/02699052.2019.1565895

15 Sandmo SB, Filipcik P, Cente M, *et al.* Neurofilament light and tau in serum after head-impact exposure in soccer. *Brain Inj* 2020;**34**:602–9. doi:10.1080/02699052.2020.1725129

16 Stålnacke BM, Sojka P. Repeatedly heading a soccer ball does not increase serum levels of S-100B, a biochemical marker of brain tissue damage: An experimental Study. *Biomark Insights* 2008;**2008**:87–91. doi:10.4137/bmi.s359

17 Straume-Naesheim TM, Andersen TE, Jochum M, *et al.* Minor head trauma in soccer and serum levels of S100B. *Neurosurgery* 2008;**62**:1297-305; discussion 1305-6.

For peer review only

doi:10.1227/01.neu.0000333301.34189.3d

18 Wirsching A, Chen Z, Bevilacqua ZW, *et al.* Association of Acute Increase in Plasma Neurofilament Light with Repetitive Subconcussive Head Impacts: A Pilot Randomized Control Trial. *J Neurotrauma* 2019;**36**:548–53. doi:10.1089/neu.2018.5836

19 Tricco AC, Lillie E, Zarin W, *et al.* PRISMA extension for scoping reviews (PRISMA-ScR): Checklist and explanation. Ann. Intern. Med. 2018;**169**:467–73. doi:10.7326/M18-0850

20 Sterne JA, Hernán MA, Reeves BC, *et al.* ROBINS-I: A tool for assessing risk of bias in non-randomised studies of interventions. *BMJ* 2016;**355**. doi:10.1136/bmj.i4919

21 Mainwaring L, Ferdinand Pennock KM, Mylabathula S, *et al.* Subconcussive head impacts in sport: A systematic review of the evidence. *Int J Psychophysiol* 2018;**132**:39–54. doi:10.1016/j.ijpsycho.2018.01.007

22 Comper P, Hutchison M, Magrys S, *et al.* Evaluating the methodological quality of sports neuropsychology concussion research: A systematic review. *Brain Inj* 2010;**24**:1257–71. doi:10.3109/02699052.2010.506854

AUTHORS' CONTRIBUTIONS

M.N., L-M.L., S.M., M.I. drafted the manuscript; L.W., A.H., T.D.V., E.S., E.D., contributed to the design of the review and approved the final version of the manuscript.

FUNDING STATEMENT

This review is supported by the University of Stirling (no grant number applies). L.W. also received support as part of Framework 7 programme of the European Union (CENTER-TBI,

For peer review only

Grant number: 602150-2). S.M. received research support from the Italian Ministry of Health (GR-2013-02354960).

COMPETING INTESRESTS STATEMENT

None of the authors have any competing interest to declare.

For peer review only

SUPPLEMENTAL MATERIAL

S1 Search strategy

Following concepts will be searched as: 1 AND 2 AND 3.

1. Sub-concussive head impacts:

(subconcussi*) OR (sub-concussi*) OR (concussi*) OR (*TBI) OR (trauma*) OR (impact*) OR (head*) OR (brain*) OR (injur*)

2. Biomarker:

(biomarker*) OR (marker*) OR (cytokines) OR (coagulation) OR (blood) OR (plasma) OR (serum) OR (“cerebrospinal fluid”) OR (CSF) OR (saliva) OR (urine) OR (neurofilament) OR (NFL) OR (S100*) OR (S-100*) OR (tau) OR (enolase) OR (NSE) OR (“glial fibrillary acidic protein”) OR (GFAP) OR (glial) OR (microRNA*) OR (miRNA*) OR (UCH-L1) OR (Ubiquitin C-terminal hydrolase L1) OR (Ubiquitin carboxyl-terminal hydrolase isozyme L1)

3. Contact sport:

(contact NEAR/1 sport*) OR (collision NEAR/1 sport*) OR (soccer) OR (football) OR (boxing) (sparr*) OR (rugby) OR (*hockey)

Appropriate MeSH terms in some databases will also be included and depending on the database minor adjustments in the search terms and/or Boolean/proximity operators will be applied.

For peer review only

S2 The Risk Of Bias In Non-randomized Studies – of Interventions (ROBINS-I) assessment tool (version for cohort-type studies)

Version 19 September 2016

This work is licensed under a Creative Commons Attribution-NonCommercial-NoDerivatives 4.0 International License.

ROBINS-I tool (Stage I): At protocol stage

Specify the review question

Participants	Male and female contact sport players (including but not limited to American football, rugby, ice-hockey, soccer, boxing) of any age and player level.
Experimental intervention	Exposure to repetitive sub-concussive head impacts (RSHI) that do not result in consequences that meet the criteria for traumatic brain injury however mild (including concussion and suspected concussion). Those impacts may be a result of either a direct head impact acquired through for example soccer heading, sparring and head-to-body collisions, or indirectly through full-body collisions between players or between player and object.
Comparator	All possible comparisons, studies with within and between groups/conditions designs are acceptable, as well as any type of control groups/conditions such as static or exercise-based control groups. Studies without control groups/conditions, as well as comparisons between exposure to high versus low number of impacts will also be included in this review.
Outcomes	The concentrations in the biofluid markers following an exposure to RSHI across groups/conditions and, where reported, the differences between the concentrations pre-to post RSHI serve as outcome measures.

List the confounding domains relevant to all or most studies

Baseline measures may be affected by exposure to head impacts (including history of concussion) and peripheral injuries prior to participation.

List co-interventions that could be different between intervention groups and that could impact on outcomes

Physical exercise during the exposure to head impacts. Peripheral injuries and impacts.

For peer review only

Preferred Reporting Items for Systematic reviews and Meta-Analyses extension for Scoping Reviews (PRISMA-ScR) Checklist

SECTION	ITEM	PRISMA-ScR CHECKLIST ITEM	REPORTED ON PAGE #
**TITLE**			
Title	1	Identify the report as a scoping review.	1
**ABSTRACT**			
Structured summary	2	Provide a structured summary that includes (as applicable): background, objectives, eligibility criteria, sources of evidence, charting methods, results, and conclusions that relate to the review questions and objectives.	2
**INTRODUCTION**			
Rationale	3	Describe the rationale for the review in the context of what is already known. Explain why the review questions/objectives lend themselves to a scoping review approach.	3 & 4
Objectives	4	Provide an explicit statement of the questions and objectives being addressed with reference to their key elements (e.g., population or participants, concepts, and context) or other relevant key elements used to conceptualize the review questions and/or objectives.	4
**METHODS**			
Protocol and registration	5	Indicate whether a review protocol exists; state if and where it can be accessed (e.g., a Web address); and if available, provide registration information, including the registration number.	N/A
Eligibility criteria	6	Specify characteristics of the sources of evidence used as eligibility criteria (e.g., years considered, language, and publication status), and provide a rationale.	4 & 5
Information sources*	7	Describe all information sources in the search (e.g., databases with dates of coverage and contact with authors to identify additional sources), as well as the date the most recent search was executed.	6 & 7

For peer review only

EFFECTS OF REPETITIVE SUB-CONCUSSIVE HEAD IMPACTS ON BIOFLUID MARKERS

SECTION	ITEM	PRISMA-ScR CHECKLIST ITEM	REPORTED ON PAGE #
Search	8	Present the full electronic search strategy for at least 1 database, including any limits used, such that it could be repeated.	S1
Selection of sources of evidence†	9	State the process for selecting sources of evidence (i.e., screening and eligibility) included in the scoping review.	5 – 7
Data charting process‡	10	Describe the methods of charting data from the included sources of evidence (e.g., calibrated forms or forms that have been tested by the team before their use, and whether data charting was done independently or in duplicate) and any processes for obtaining and confirming data from investigators.	7 & 8
Data items	11	List and define all variables for which data were sought and any assumptions and simplifications made.	N/A
Critical appraisal of individual sources of evidence§	12	If done, provide a rationale for conducting a critical appraisal of included sources of evidence; describe the methods used and how this information was used in any data synthesis (if appropriate).	8
Synthesis of results	13	Describe the methods of handling and summarizing the data that were charted.	8
**RESULTS**			
Selection of sources of evidence	14	Give numbers of sources of evidence screened, assessed for eligibility, and included in the review, with reasons for exclusions at each stage, ideally using a flow diagram.	N/A
Characteristics of sources of evidence	15	For each source of evidence, present characteristics for which data were charted and provide the citations.	N/A
Critical appraisal within sources of evidence	16	If done, present data on critical appraisal of included sources of evidence (see item 12).	N/A
Results of individual sources of evidence	17	For each included source of evidence, present the relevant data that were charted that relate to the review questions and objectives.	N/A

For peer review only

EFFECTS OF REPETITIVE SUB-CONCUSSIVE HEAD IMPACTS ON BIOFLUID MARKERS

SECTION	ITEM	PRISMA-ScR CHECKLIST ITEM	REPORTED ON PAGE #
Synthesis of results	18	Summarize and/or present the charting results as they relate to the review questions and objectives.	N/A
**DISCUSSION**			
Summary of evidence	19	Summarize the main results (including an overview of concepts, themes, and types of evidence available), link to the review questions and objectives, and consider the relevance to key groups.	9
Limitations	20	Discuss the limitations of the scoping review process.	3
Conclusions	21	Provide a general interpretation of the results with respect to the review questions and objectives, as well as potential implications and/or next steps.	N/A
**FUNDING**			
Funding	22	Describe sources of funding for the included sources of evidence, as well as sources of funding for the scoping review. Describe the role of the funders of the scoping review.	12

JBI = Joanna Briggs Institute; PRISMA-ScR = Preferred Reporting Items for Systematic reviews and Meta-Analyses extension for Scoping Reviews.

* Where *sources of evidence* (see second footnote) are compiled from, such as bibliographic databases, social media platforms, and Web sites.

† A more inclusive/heterogeneous term used to account for the different types of evidence or data sources (e.g., quantitative and/or qualitative research, expert opinion, and policy documents) that may be eligible in a scoping review as opposed to only studies. This is not to be confused with *information sources* (see first footnote).

‡ The frameworks by Arksey and O'Malley (6) and Levac and colleagues (7) and the JBI guidance (4, 5) refer to the process of data extraction in a scoping review as data charting.

§ The process of systematically examining research evidence to assess its validity, results, and relevance before using it to inform a decision. This term is used for items 12 and 19 instead of "risk of bias" (which is more applicable to systematic reviews of interventions) to include and acknowledge the various sources of evidence that may be used in a scoping review (e.g., quantitative and/or qualitative research, expert opinion, and policy document).

From: Tricco AC, Lillie E, Zarin W, et al. PRISMA Extension for Scoping Reviews (PRISMAScR): Checklist and Explanation. Ann Intern. Med. 2018;169:467–73. doi: 10.7326/M18-0850

For peer review only

BMJ Open

Effects of Sport-related Repetitive Sub-concussive Head Impacts on Biofluid Markers: A Scoping Review Protocol

Journal:	*BMJ Open*
Manuscript ID	bmjopen-2020-046452.R1
Article Type:	Protocol
Date Submitted by the Author:	03-Apr-2021
Complete List of Authors:	Lember, Liivia-Mari; University of Stirling Faculty of Natural Sciences, Department of Psychology<br>Ntikas, Michail; University of Stirling Faculty of Natural Sciences, Department of Psychology<br>Mondello, Stefania; University of Messina Faculty of Medicine and Surgery, Biomedical and Dental Sciences and Morphofunctional Imaging<br>Wilson, Lindsay; University of Stirling Department of Psychology,<br>Hunter, Angus; University of Stirling Faculty of Health Sciences and Sport, Physiology Exercise and Nutrition Research Group<br>Di Virgilio, Thomas; University of Stirling Faculty of Health Sciences and Sport, Physiology Exercise and Nutrition Research Group<br>Santoro, Emanuela; University of Messina Faculty of Medicine and Surgery, Biomedical and Dental Sciences and Morphofunctional Imaging<br>Ietswaart, Magdalena; University of Stirling Faculty of Natural Sciences, Department of Psychology
**Primary Subject Heading**:	Neurology
Secondary Subject Heading:	Sports and exercise medicine
Keywords:	Neurological injury < NEUROLOGY, Neuropathology < NEUROLOGY, SPORTS MEDICINE

SCHOLARONE™
Manuscripts

I, the Submitting Author has the right to grant and does grant on behalf of all authors of the Work (as defined in the below author licence), an exclusive licence and/or a non-exclusive licence for contributions from authors who are: i) UK Crown employees; ii) where BMJ has agreed a CC-BY licence shall apply, and/or iii) in accordance with the terms applicable for US Federal Government officers or employees acting as part of their official duties; on a worldwide, perpetual, irrevocable, royalty-free basis to BMJ Publishing Group Ltd ("BMJ") its licensees and where the relevant Journal is co-owned by BMJ to the co-owners of the Journal, to publish the Work in this journal and any other BMJ products and to exploit all rights, as set out in our licence.

The Submitting Author accepts and understands that any supply made under these terms is made by BMJ to the Submitting Author unless you are acting as an employee on behalf of your employer or a postgraduate student of an affiliated institution which is paying any applicable article publishing charge ("APC") for Open Access articles. Where the Submitting Author wishes to make the Work available on an Open Access basis (and intends to pay the relevant APC), the terms of reuse of such Open Access shall be governed by a Creative Commons licence – details of these licences and which Creative Commons licence will apply to this Work are set out in our licence referred to above.

Other than as permitted in any relevant BMJ Author's Self Archiving Policies, I confirm this Work has not been accepted for publication elsewhere, is not being considered for publication elsewhere and does not duplicate material already published. I confirm all authors consent to publication of this Work and authorise the granting of this licence.

For peer review only

Effects of Sport-related Repetitive Sub-concussive Head Impacts on Biofluid Markers: A Scoping Review Protocol

Liivia-Mari Lember*[1,2], Michail Ntikas*[1], Stefania Mondello[3], Lindsay Wilson[1], Angus Hunter[2], Thomas Di Virgilio[2], Emanuela Santoro[3] and Magdalena Ietswaart[1]

1. Department of Psychology, University of Stirling, Scotland, UK
2. Physiology Exercise and Nutrition Research Group, University of Stirling, Scotland, UK
3. Biomedical and Dental Sciences and Morphofunctional Imaging, University of Messina, Italy

* joint first author

Corresponding author: Magdalena Ietswaart

Department of Psychology, University of Stirling

Stirling, FK9 4LA, United Kingdom

Email: magdalena.ietswaart@stir.ac.uk

Phone: + 44 (0) 7711001106

Word count: 1818

Keywords: scoping review, biomarkers, subconcussive head impacts, brain injury, diagnosis, sport

For peer review only

ABSTRACT

Introduction: Repetitive sub-concussive sport-related head impacts (RSHI) are increasingly thought to be associated with adverse long-term outcomes. However, owing to potentially subtle effects, accurate assessment of harm to the brain as a consequence of RSHI is a major challenge and an unmet need. Several studies suggest that biofluid markers can be valuable objective tools to aid the diagnosis and injury characterization and help in medical decision-making. Still, by and large, the results have been limited, heterogeneous and inconsistent. The main aims of this scoping review are therefore (1) to systematically examine the extent, nature, and quality of available evidence from studies investigating effects of RSHI on biomarkers, and (2) to formulate guidelines and identify gaps with the aim to inform future clinical studies and the development of research priorities.

Methods and analyses: We will use a comprehensive search strategy to retrieve all available and relevant articles in the literature. The following electronic databases will be systematically searched: MEDLINE (EBSCO host); (from 1809 to 2020); Scopus (from 1788 to 2020); SPORTDiscus (from 1892 to 2020); CINAHL Complete (from 1937 to 2020); PsycINFO (from 1887 to 2020); Cochrane Library (to 2020); OpenGrey (to 2020); ClinicalTrials.gov (to 2020); and World Health Organization International Clinical Trials Registry Platform (to 2020). We will consider primarily biomedical studies evaluating the biofluid markers following RSHI. Two independent reviewers will screen articles for inclusion using predefined eligibility criteria and extract data of retained articles. Disagreements will be resolved through consensus or arbitrated by a third reviewer if necessary. Data will be reported qualitatively given the heterogeneity of the included studies. In synthesizing the evidence, we will structure results by markers, sample types, outcomes, sport, and timepoints.

For peer review only

46 **Ethics and dissemination:** Ethics approval is not required. We will submit results for peer-review publication, and present at relevant conferences.

For peer review only

STRENGTHS AND LIMITATIONS OF THIS STUDY

- To our knowledge, this scoping review is the first review to systematically and comprehensively map the existing evidence of the effects of RSHI on biofluid markers.
- We employ broad inclusion criteria to ensure that relevant studies are not missed.
- Results from our study are designed to advance the understanding of the effects of repetitive sub-concussive head impacts on neurobiochemical markers and provide guidelines for future clinical research.
- A potential limitation may be a lack of a universally accepted and standardized definition of what sub-concussive head impacts are, which may introduce increased subjective judgement with regards to inclusion of studies.

INTRODUCTION

Unlike concussion, sub-concussive head impacts do not frequently elicit overt symptoms and, therefore, are often regarded as benign. Nonetheless, there is emerging evidence that both concussive and sub-concussive impacts result in structural and functional changes in the brain,[1,2] representing a potential contributing factor to long-term cognitive sequalae and/or neurodegenerative disease.[3–5] This raises concerns for athletes participating in contact sports who routinely experience repetitive head impacts, particularly owing to the challenge to accurately detect resulting subtle brain changes.

Measurable biomarkers in blood have been found to significantly increase following traumatic brain injury (TBI),[6–8] and be correlated with injury severity and outcomes.[9,10] Hence, they might be a helpful diagnostic tool,[11,12] capable of informing management of athletes. However, studies evaluating biomarker concentrations following repetitive sub-

For peer review only - http://bmjopen.bmj.com/site/about/guidelines.xhtml

concussive head impacts (RSHI) such as in contact sport athletes have yielded mixed results.[13–19] These contrasting findings may be caused by methodological and analytical variability (e.g. diverse settings, sampling times and types, study designs) as well as the lack of a universally accepted and standardized definition of RSHI. For the purposes of this review, RSHI are operationally defined as routine repetitive intentional or unintentional non-concussive head impacts acquired during contact sport participation. On completion of this scoping review the aim is to provide a comprehensive evidence-based operational definition of RSHI to be adopted in future work.

The use of biofluid markers for detection of brain changes following RSHI is an emerging field of research. As such, we considered that at this stage a scoping review comprehensively mapping the studies from different sports applying various designs will be more advantageous compared to a systematic review that would be restricted to only part of the existing literature.[20,21] Therefore, the aim of this review is to systematically scope the existing body of evidence, evaluate the quality and the adequacy of reporting, and identify research gaps to guide future research to support the clinical utility of biomarkers for RSHI.

Objectives

The primary aim is to systematically examine the extent, nature, and quality of available studies that have investigated the effects of RSHI on biofluid brain injury markers. The secondary aim is to reach an informed view of an acceptable set of features for future work concerning RSHI, formulate guidelines for future research and identify literature gaps to inform future clinical studies and the development of research priorities. An additional aim is to assess the feasibility of

For peer review only

conducting systematic review and meta-analysis investigating the effects of RSHI on biofluid markers.

METHODS

In this scoping review, we will use a systematic and comprehensive approach to retrieve all studies published in peer reviewed academic journals, along with registered clinical trials and grey literature. Our work will adhere to the PRISMA extension for scoping reviews.[22]

Study Eligibility

This review will include studies where acute or chronic exposure to repetitive sub-concussive sport-related head impacts occurred and where biofluid markers were assessed (including, but not limited to, S-100B, NF-L, T-tau, NSE, GFAP, microRNA). Studies with the primary aim to assess the effect of RSHI on biofluid markers will be included if there is evidence of RSHI exposure. This evidence could be minutes of boxing training, years of soccer playing, boxing/rugby matches played and so on. Studies primarily focused on concussion in which contact sport control groups were used, will be included if there is sufficient evidence of sub-concussive head impact exposure in the control group (impact data, video recording) and biofluid marker samples were taken before and after this exposure, thus making it a valid experimental group for assessing RSHI.

This review will not include studies assessing biomarker concentrations following solely sports-related concussion or traumatic brain injury or studies assessing biofluid markers of peripheral injury. We will also exclude any studies with potentially overlapping population/biomarker data, and review articles.

For peer review only

We will not place any restrictions on methodological standards, design and sample size. Studies will be included regardless of geographic location and date of publication. We will be examining reports in the English, French, German, and Italian languages.

PECOS Criteria

We will include clinical studies if they contain the PECOS criteria outlined below:

Types of Participants

The population of interest in the current review are active or retired male and female contact sport players (including but not limited to American football, rugby, ice-hockey, soccer, boxing) of any age and player level.

Types of Interventions/Exposure

Acute or chronic exposure to RSHI. Those impacts may be a result of either a direct head impact acquired through for example soccer heading, sparring and head-to-body collisions, or indirectly through full-body collisions between players or between player and object.

Types of Comparisons

We will include all possible comparisons; studies with within and between groups/conditions designs are acceptable, as well as any type of control groups/conditions such as static or exercise-based control groups. Studies without control groups/conditions, as well as comparisons between exposure to high versus low number of impacts will also be included in this scoping review.

For peer review only

140

141 Type of Outcome Measures

142 The concentrations of biofluid markers following acute or chronic exposure to RSHI across
143 groups/conditions and, where reported, the differences between the concentrations pre-to post
144 RSHI serve as outcome measures.

145

146 Setting

147 We will include all relevant settings (for example field and lab-based studies).

148

149 **Search Strategy**

150 An electronic search will be carried out in the following databases: Cochrane Library,
151 CINAHL Complete, PsycINFO, MEDLINE (EBSCO host), Scopus, SPORTDiscus, OpenGrey.
152 The following databases will be searched for ongoing registered clinical trials: ClinicalTrials.gov,
153 World Health Organization International Clinical Trials Registry Platform. Reference lists of
154 included studies will also be searched.

155 Key descriptors that include terms for Sub-concussive head impacts, Biomarker, Contact
156 sport, will be used for the search (see table 1).

157

158 **Table 1.** Search strategy including the three concepts and corresponding keywords.

**Concept**	**Keywords**
Sub -concussive head impacts	(subconcussi*) OR (sub-concussi*) OR (concussi*) OR (*TBI) OR (trauma*) OR (impact*) OR (head*) OR (brain*) OR (injur*)
Biomarker	(biomarker*) OR (marker*) OR (cytokines) OR (coagulation) OR (blood) OR (plasma) OR (serum) OR ("cerebrospinal fluid") OR (CSF) OR (saliva) OR (urine) OR (neurofilament) OR (NFL) OR (S100*) OR (S -100*) OR (tau) OR (enolase) OR (NSE) OR

For peer review only

	(“glial fibrillary acidic protein”) OR (GFAP) OR (glial) OR (microRNA*) OR (miRNA*) OR (UCH -L1) OR (Ubiquitin C -terminal hydrolase L1) OR (Ubiquitin carboxyl -terminal hydrolase isozyme L1)
Contact sport	(contact NEAR/1 sport*) OR (collision NEAR/1 sport*) OR (soccer) OR (football) OR (boxing) (sparr*) OR (rugby) OR (*hockey)

Concepts will be separated with the Boolean operator ‘AND’. Depending on the database minor adjustments in keywords and proximity operators will be applied.

Study Selection

After removal of duplicates, two reviewers will independently screen the titles and abstracts against the eligibility criteria. Any disputes between reviewers will be resolved through discussion and if necessary, by a third member. The same process will be repeated on the full text to confirm inclusion in the scoping review. Studies excluded during the full-text screening will be attached with exclusion reason(s) as an appendix. The exclusion reason(s) will inform whether the 3rd judgment was employed.

Data Extraction

Data will be recorded independently by two reviewers using a standardized data collection form. Disagreements will be discussed until consensus is reached and, if necessary, a third reviewer will be consulted for arbitration. The following information will be extracted if possible: (1) First author, (2) Year of publication, (3) Title, (4) Study aim(s), (5) Study design, (6) Participant characteristics (sample size, age, sex, sport, exclusion/inclusion criteria), (7) Control group (within or between group/condition comparisons; control group characteristics – static, exercise, no impacts, some impacts but less than in the exposed group etc.), (8) Setting (laboratory based or field study), (9) Characteristics of biomarkers (biomarker(s) investigated, time of sampling,

For peer review only

sampling source [plasma/serum/CSF/saliva], levels [mean ± SD or median with IQR], any other data deemed relevant), (10) Laboratory aspects (type of assay used, limit of detection, limit of quantitation, sampling to freezing time), (11) Impact data (i.e., source of impacts [e.g. football heading] with the number of impacts, and linear and rotational acceleration [mean ± SD or median with IQR], (12) Method for impact recording (i.e. accelerometer and gyroscope, video footage, self-report, estimation based on previous literature), (13) Outcome measures and findings (e.g. cognitive or brain-related clinical measures), and (14) Study limitations.

Risk of Bias and Quality Assessment of Included Studies

A modified version of ROBINS-I risk of bias assessment will be used in this review.[23] Moreover, a modified version of the Subconcussion-Specific Tool (SST) will be used to assess the quality of the included studies.[2,24] Even though performing a risk of bias and quality assessment is not a prerequisite for a scoping review, its use will help improve the quality of this review and help determine acceptable features in the research domain of RSHI. Further, the risk of bias and quality assessment can potentially provide evidence for the feasibility of future systematic review. ROBINS-I tool protocol stage is included in the supplementary material (S1).

Data Analysis

Studies will be grouped by the type of study (acute or chronic), biomarker, and sport. Study findings, designs, populations, control groups, sources and amount of head impact exposure, sampling methods and setting will be summarised. Methodological differences, gaps in the literature and quality assessment of the included studies will be analysed to provide implications

For peer review only

for future research, and to determine the advantages and practicability of performing a further systematic review.

Patient and public involvement

No new patients will be enrolled as part of this investigation.

DISCUSSION

This scoping review will provide an overview of the existing body of evidence of the effect of sport-related repetitive sub-concussive head impacts on biofluid markers. Further, the quality and limitations of the available evidence will be assessed to aid the development of high-quality future clinical studies by providing guidelines that will increase future studies' clinical utility and homogeneity. Moreover, the findings of this review will inform the decision as to whether performing systematic review is feasible based on the existing body of evidence.

A strength of this review will be the comprehensive inclusion criteria and search strategy. However, the lack of a universally accepted and standardized definition of repetitive sub-concussive head impacts may introduce ambiguity in the inclusion of studies. To minimize the risk of potential differences in opinion, the current protocol has outlined the types of exposure that will be considered to qualify as repetitive sub-concussive head impacts. Further, we have designated a member of the research team to resolve any disputes that may incur. Moreover, the included studies will be assessed for quality, and limitations will be recorded. Reasons will be noted for studies that are excluded during the full-text screening.

While the broad nature of scoping reviews will not allow for quantitative analysis,[21,22] the findings from this work will be advantageous for future research by identifying the knowledge

For peer review only

gaps and limitations within the emerging field of the effect of sport-related repetitive head impacts on biofluid markers.

ETHICS AND DISSEMINATION

Ethics approval for this scoping review is not required. The findings will be published in a peer-reviewed journal and presented at relevant conferences, such as the Symposia of the National and International Neurotrauma Societies and the Neurocritical Care Society Meeting, and university and stakeholder workshops. Other forms of dissemination will include academic theses.

REFERENCES

1 Di Virgilio TG, Hunter A, Wilson L, *et al.* Evidence for Acute Electrophysiological and Cognitive Changes Following Routine Soccer Heading. *EBioMedicine* 2016;**13**:66–71. doi:10.1016/j.ebiom.2016.10.029

2 Mainwaring L, Ferdinand Pennock KM, Mylabathula S, *et al.* Subconcussive head impacts in sport: A systematic review of the evidence. *Int J Psychophysiol* 2018;**132**:39–54. doi:10.1016/j.ijpsycho.2018.01.007

3 Maas AIR, Menon DK, Adelson PD, *et al.* Traumatic brain injury: integrated approaches to improve prevention, clinical care, and research. *Lancet Neurol* 2017;**16**:987–1048. doi:10.1016/S1474-4422(17)30371-X

4 McKee AC, Daneshvar DH, Alvarez VE, *et al.* The neuropathology of sport. *Acta Neuropathol* 2014;**127**:29–51. doi:10.1007/s00401-013-1230-6

5 Wilson L, Stewart W, Dams-O'Connor K, *et al.* The chronic and evolving neurological consequences of traumatic brain injury. *Lancet Neurol* 2017;**16**:813–25.

For peer review only

doi:10.1016/S1474-4422(17)30279-X

6 Herrmann M, Curio N, Jost S, *et al.* Protein S-100B and neuron specific enolase as early neurobiochemical markers of the severity of traumatic brain injury. *Restor Neurol Neurosci* 1999;**14**:109–14.

7 Shahim P, Tegner Y, Wilson DH, *et al.* Blood biomarkers for brain injury in concussed professional ice hockey players. *JAMA Neurol* 2014;**71**:684–92. doi:10.1001/jamaneurol.2014.367

8 Shahim P, Tegner Y, Marklund N, *et al.* Neurofilament light and tau as blood biomarkers for sports-related concussion. *Neurology* 2018;**90**:E1780–8. doi:10.1212/WNL.0000000000005518

9 Mondello S, Guedes VA, Lai C, *et al.* Sex Differences in Circulating T-Tau Trajectories After Sports-Concussion and Correlation With Outcome. *Front Neurol* 2020;**11**:1–8. doi:10.3389/fneur.2020.00651

10 Mondello S, Guedes VA, Lai C, *et al.* Circulating Brain Injury Exosomal Proteins following Moderate-To-Severe Traumatic Brain Injury: Temporal Profile, Outcome Prediction and Therapy Implications. *Cells* 2020;**9**:1–12. doi:10.3390/cells9040977

11 Gill J, Latour L, Diaz-Arrastia R, *et al.* Glial fibrillary acidic protein elevations relate to neuroimaging abnormalities after mild TBI. *Neurology* 2018;**91**:E1385–9. doi:10.1212/WNL.0000000000006321

12 Mondello S, Sorinola A, Czeiter E, *et al.* Blood-Based Protein Biomarkers for the Management of Traumatic Brain Injuries in Adults Presenting to Emergency Departments with Mild Brain Injury: A Living Systematic Review and Meta-Analysis. *J Neurotrauma* 2018;**21**:1–21. doi:10.1089/neu.2017.5182

For peer review only

13 Dorminy M, Hoogeveen A, Tierney RT, *et al.* Effect of soccer heading ball speed on S100B, sideline concussion assessments and head impact kinematics. *Brain Inj* 2015;**29**:1158-1164. doi:10.3109/02699052.2015.1035324

14 Kawata K, Rubin LH, Wesley L, *et al.* Acute Changes in Plasma Total Tau Levels Are Independent of Subconcussive Head Impacts in College Football Players. *J Neurotrauma* 2018;**35**:260–6. doi:10.1089/neu.2017.5376

15 Rubin LH, Tierney R, Kawata K, *et al.* NFL blood levels are moderated by subconcussive impacts in a cohort of college football players. *Brain Inj* 2019;**33**:456–62. doi:10.1080/02699052.2019.1565895

16 Sandmo SB, Filipcik P, Cente M, *et al.* Neurofilament light and tau in serum after head-impact exposure in soccer. *Brain Inj* 2020;**34**:602–9. doi:10.1080/02699052.2020.1725129

17 Stålnacke BM, Sojka P. Repeatedly heading a soccer ball does not increase serum levels of S-100B, a biochemical marker of brain tissue damage: An experimental Study. *Biomark Insights* 2008;**2008**:87–91. doi:10.4137/bmi.s359

18 Straume-Naesheim TM, Andersen TE, Jochum M, *et al.* Minor head trauma in soccer and serum levels of S100B. *Neurosurgery* 2008;**62**:1297-305; discussion 1305-6. doi:10.1227/01.neu.0000333301.34189.3d

19 Wirsching A, Chen Z, Bevilacqua ZW, *et al.* Association of Acute Increase in Plasma Neurofilament Light with Repetitive Subconcussive Head Impacts: A Pilot Randomized Control Trial. *J Neurotrauma* 2019;**36**:548–53. doi:10.1089/neu.2018.5836

20 Munn Z, Peters MDJ, Stern C, *et al.* Systematic review or scoping review? Guidance for authors when choosing between a systematic or scoping review approach. *BMC Med Res*

For peer review only

Methodol 2018;**18**:1–7. doi:10.1186/s12874-018-0611-x

21 Peters MDJ, Godfrey CM, Khalil H, *et al.* Guidance for conducting systematic scoping reviews. *Int J Evid Based Healthc* 2015;**13**:141–6. doi:10.1097/XEB.0000000000000050

22 Tricco AC, Lillie E, Zarin W, *et al.* PRISMA extension for scoping reviews (PRISMA-ScR): Checklist and explanation. Ann. Intern. Med. 2018;**169**:467–73. doi:10.7326/M18-0850

23 Sterne JA, Hernán MA, Reeves BC, *et al.* ROBINS-I: A tool for assessing risk of bias in non-randomised studies of interventions. *BMJ* 2016;**355**. doi:10.1136/bmj.i4919

24 Comper P, Hutchison M, Magrys S, *et al.* Evaluating the methodological quality of sports neuropsychology concussion research: A systematic review. *Brain Inj* 2010;**24**:1257–71. doi:10.3109/02699052.2010.506854

AUTHORS' CONTRIBUTIONS

M.N., L-M.L., S.M., M.I. drafted the manuscript; L.W., A.H., T.D.V., E.S., contributed to the design of the review and approved the final version of the manuscript.

FUNDING STATEMENT

This review is supported by the University of Stirling (no grant number applies). L.W. also received support as part of Framework 7 programme of the European Union (CENTER-TBI, Grant number: 602150-2). S.M. received research support from the Italian Ministry of Health (GR-2013-02354960).

COMPETING INTESRESTS STATEMENT

For peer review only

None of the authors have any competing interest to declare.

For peer review only

SUPPLEMENTAL MATERIAL

S1 The Risk Of Bias In Non-randomized Studies – of Interventions (ROBINS-I) assessment tool (version for cohort-type studies)

Version 19 September 2016

This work is licensed under a Creative Commons Attribution-NonCommercial-NoDerivatives 4.0 International License.

ROBINS-I tool (Stage I): At protocol stage

Specify the review question

Participants	Male and female contact sport players (including but not limited to American football, rugby, ice-hockey, soccer, boxing) of any age and player level.
Experimental intervention	Exposure to repetitive sub-concussive head impacts (RSHI) that do not result in consequences that meet the criteria for traumatic brain injury however mild (including concussion and suspected concussion). Those impacts may be a result of either a direct head impact acquired through for example soccer heading, sparring and head-to-body collisions, or indirectly through full-body collisions between players or between player and object.
Comparator	All possible comparisons, studies with within and between groups/conditions designs are acceptable, as well as any type of control groups/conditions such as static or exercise-based control groups. Studies without control groups/conditions, as well as comparisons between exposure to high versus low number of impacts will also be included in this review.
Outcomes	The concentrations in the biofluid markers following an exposure to RSHI across groups/conditions and, where reported, the differences between the concentrations pre-to post RSHI serve as outcome measures.

List the confounding domains relevant to all or most studies

Baseline measures may be affected by sex, central nervous system disease, exposure to head impacts (including history of concussion) and peripheral injuries prior to participation.

List co-interventions that could be different between intervention groups and that could impact on outcomes

Physical exercise during the exposure to head impacts. Peripheral injuries and impacts.

For peer review only

Preferred Reporting Items for Systematic reviews and Meta-Analyses extension for Scoping Reviews (PRISMA-ScR) Checklist

SECTION	ITEM	PRISMA-ScR CHECKLIST ITEM	REPORTED ON PAGE #
**TITLE**			
Title	1	Identify the report as a scoping review.	1
**ABSTRACT**			
Structured summary	2	Provide a structured summary that includes (as applicable): background, objectives, eligibility criteria, sources of evidence, charting methods, results, and conclusions that relate to the review questions and objectives.	2 & 3
**INTRODUCTION**			
Rationale	3	Describe the rationale for the review in the context of what is already known. Explain why the review questions/objectives lend themselves to a scoping review approach.	3 & 4
Objectives	4	Provide an explicit statement of the questions and objectives being addressed with reference to their key elements (e.g., population or participants, concepts, and context) or other relevant key elements used to conceptualize the review questions and/or objectives.	5 & 6
**METHODS**			
Protocol and registration	5	Indicate whether a review protocol exists; state if and where it can be accessed (e.g., a Web address); and if available, provide registration information, including the registration number.	N/A
Eligibility criteria	6	Specify characteristics of the sources of evidence used as eligibility criteria (e.g., years considered, language, and publication status), and provide a rationale.	6 & 7
Information sources*	7	Describe all information sources in the search (e.g., databases with dates of coverage and contact with authors to identify additional sources), as well as the date the most recent search was executed.	8

For peer review only

SECTION	ITEM	PRISMA-ScR CHECKLIST ITEM	REPORTED ON PAGE #
Search	8	Present the full electronic search strategy for at least 1 database, including any limits used, such that it could be repeated.	8 & 9
Selection of sources of evidence†	9	State the process for selecting sources of evidence (i.e., screening and eligibility) included in the scoping review.	7 – 9
Data charting process‡	10	Describe the methods of charting data from the included sources of evidence (e.g., calibrated forms or forms that have been tested by the team before their use, and whether data charting was done independently or in duplicate) and any processes for obtaining and confirming data from investigators.	9 & 10
Data items	11	List and define all variables for which data were sought and any assumptions and simplifications made.	N/A
Critical appraisal of individual sources of evidence§	12	If done, provide a rationale for conducting a critical appraisal of included sources of evidence; describe the methods used and how this information was used in any data synthesis (if appropriate).	10
Synthesis of results	13	Describe the methods of handling and summarizing the data that were charted.	10 & 11
**RESULTS**			
Selection of sources of evidence	14	Give numbers of sources of evidence screened, assessed for eligibility, and included in the review, with reasons for exclusions at each stage, ideally using a flow diagram.	N/A
Characteristics of sources of evidence	15	For each source of evidence, present characteristics for which data were charted and provide the citations.	N/A
Critical appraisal within sources of evidence	16	If done, present data on critical appraisal of included sources of evidence (see item 12).	N/A
Results of individual sources of evidence	17	For each included source of evidence, present the relevant data that were charted that relate to the review questions and objectives.	N/A

For peer review only

EFFECTS OF REPETITIVE SUB-CONCUSSIVE HEAD IMPACTS ON BIOFLUID MARKERS

SECTION	ITEM	PRISMA-ScR CHECKLIST ITEM	REPORTED ON PAGE #
Synthesis of results	18	Summarize and/or present the charting results as they relate to the review questions and objectives.	N/A
**DISCUSSION**			
Summary of evidence	19	Summarize the main results (including an overview of concepts, themes, and types of evidence available), link to the review questions and objectives, and consider the relevance to key groups.	11 & 12
Limitations	20	Discuss the limitations of the scoping review process.	4, 11 & 12
Conclusions	21	Provide a general interpretation of the results with respect to the review questions and objectives, as well as potential implications and/or next steps.	N/A
**FUNDING**			
Funding	22	Describe sources of funding for the included sources of evidence, as well as sources of funding for the scoping review. Describe the role of the funders of the scoping review.	15

JBI = Joanna Briggs Institute; PRISMA-ScR = Preferred Reporting Items for Systematic reviews and Meta-Analyses extension for Scoping Reviews.

* Where *sources of evidence* (see second footnote) are compiled from, such as bibliographic databases, social media platforms, and Web sites.

† A more inclusive/heterogeneous term used to account for the different types of evidence or data sources (e.g., quantitative and/or qualitative research, expert opinion, and policy documents) that may be eligible in a scoping review as opposed to only studies. This is not to be confused with *information sources* (see first footnote).

‡ The frameworks by Arksey and O'Malley (6) and Levac and colleagues (7) and the JBI guidance (4, 5) refer to the process of data extraction in a scoping review as data charting.

§ The process of systematically examining research evidence to assess its validity, results, and relevance before using it to inform a decision. This term is used for items 12 and 19 instead of "risk of bias" (which is more applicable to systematic reviews of interventions) to include and acknowledge the various sources of evidence that may be used in a scoping review (e.g., quantitative and/or qualitative research, expert opinion, and policy document).

From: Tricco AC, Lillie E, Zarin W, et al. PRISMA Extension for Scoping Reviews (PRISMAScR): Checklist and Explanation. Ann Intern. Med. 2018;169:467–73. doi: 10.7326/M18-0850

For peer review only
